# A novel synthesis, X-ray analysis and computational studies of (Z)-ethyl 2-((Z)-5-((dimethylamino)methylene)- 4-oxo-3-phenylthiazolidin-2-ylidene)acetate as a potential anticancer agent

**DOI:** 10.1186/s13065-019-0554-2

**Published:** 2019-03-26

**Authors:** Yahia N. Mabkhot, Mohammed M. Alharbi, Salim. S. Al-Showiman, Saied M. Soliman, Nabila A. Kheder, Wolfgang Frey, Abdulrhman Asayari, Abdullatif Bin Muhsinah, H. Algarni

**Affiliations:** 1 Department of Pharmaceutical Chemistry, College of Pharmacy, King Khalid University, Abha, 61441 Saudi Arabia; 2 Department of Chemistry, College of Science, King Saud University, P. O. Box 2455, Riyadh, 11451 Saudi Arabia; 3 Department of Chemistry, Faculty of Science, Alexandria University, P.O. Box 426, Alexandria, Ibrahimia-21321 Egypt; 4 Department of Chemistry, Rabigh College of Science and Art, King Abdulaziz University, Jeddah, Saudi Arabia; 5 Department of Chemistry, Faculty of Science, Cairo University, Giza, 12613 Egypt; 6 Institut für OrganischeChemie, Universitӓt Stuttgart, Pfaffenwaldring 55, 70569 Stuttgart, Germany; 7 Department of Pharmacognosy, College of Pharmacy, King Khalid University, Abha, 61441 Saudi Arabia; 8 Department of Physics, Faculty of Sciences, King Khalid University, P. O. Box 9004, Abha, Saudi Arabia; 9 Research Centre for Advanced Materials Science (RCAMS), King Khalid University, P. O. Box 9004, Abha, 61413 Saudi Arabia

**Keywords:** Thiazolidinone, X-ray crystallography, Computational studies, DMF-DMA, Cytotoxic activity

## Abstract

**Background:**

4-Thiazolidinone ring is reported to have almost all types of biological activities. Also, it present in many marketed drugs.

**Results:**

Ethyl acetoacetate reacted with phenyl isothiocyanate and ethyl chloroacetate in presence of K_2_CO_3_ and DMF to afford the thiazolidinone derivative **5**. Thiazolidinone **5** reacted with dimethylformamide-dimethylacetal to afford (Z)-ethyl 2-((Z)-5-((dimethylamino) methylene)-4-oxo-3-phenylthiazolidin-2-ylidene)acetate (**6**). The structure of thiazolidinone **6** was elucidated from its spectral analysis and X-ray crystallography and was optimized using B3LYP/6-31G(d,p) method. The geometric parameters and NMR spectra were discussed both experimentally and theoretically. Also, the natural charges at the different atomic sites were predicted. The synthesized compounds had moderate cytotoxic activity.

**Conclusions:**

An unexpected synthesis of (Z)-ethyl 2-((Z)-5-((dimethylamino)methylene)-4-oxo-3-phenylthiazolidin-2-ylidene)acetate via deacetylation mechanism. The structure was established using X-ray and spectral analysis. The geometric parameters, and NMR spectra were discussed. The synthesized compounds showed moderate anticancer activity. 
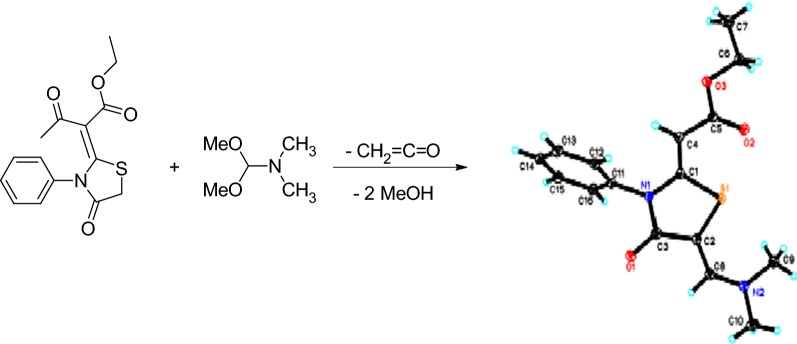

## Introduction

**Fig. 1:**
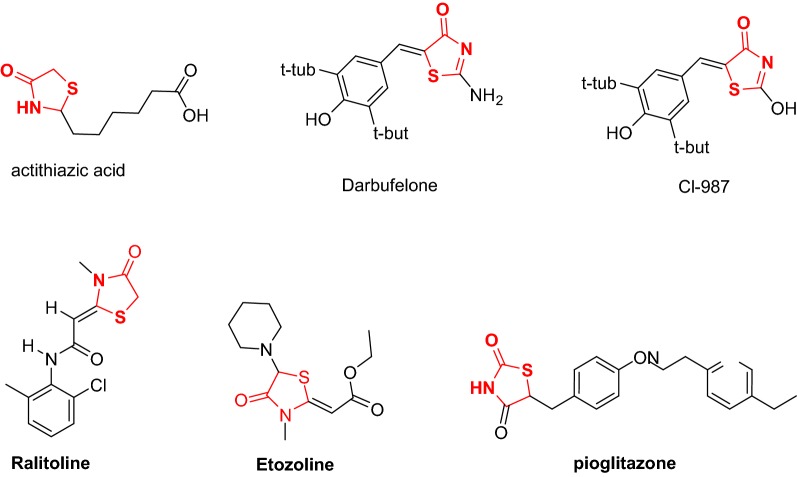
Examples of some drugs containing 4-thiazolidinone ring

The thiazolidinone ring had diverse biological activities such as antimycobacterial [1], antimicrobial [2], anticancer [3], anticonvulsant [4], antiparasitic [5], antidiabetic [6], and antihypertensive [7]. Also, many clinically used drugs contain thiazolidinone ring in their skeletons such as antibiotic actithiazic acid [8], dual COX/LOX inhibitors (Darbufelone and CI-987) [9], Ralitoline, Etozoline, and Pioglitazone (Fig. 1).

Due to this diversity in the biological activities of 4-thiazolidinones, there are several procedures have been reported for their synthesis [10–14]. In this research, we synthesize new 4-thiazolidinone derivative 6 in a pure stat, also, we compare cytotoxic activity of synthesized compounds with standard anticancer drug Vinblastine against the colon carcinoma (HCT-116) cell line using MTT assay.

## Results and discussion

### Chemistry

**Scheme 1:**
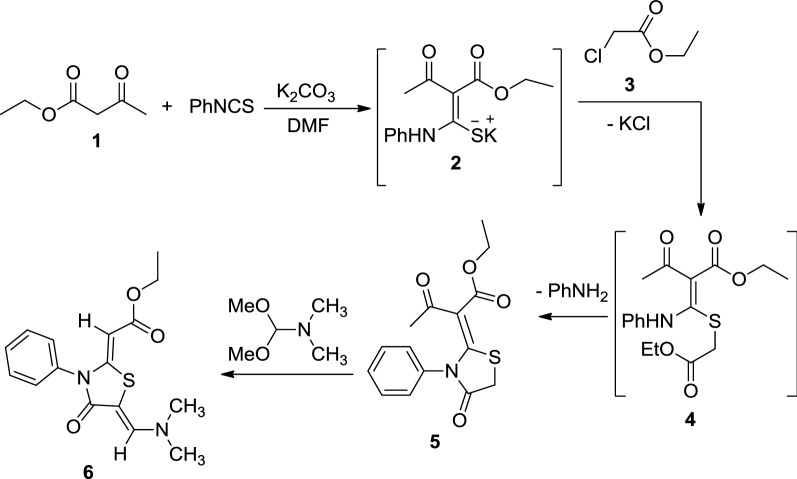
Synthesis of thiazolidinones **5** and **6**

The thiazolidinone derivative **5** was prepared according to the reported method [15]. Refluxing of the thiazolidinone **5** with dimethylformamide–dimethylacetal in DMF gave only one isolable product, the product of this reaction is (Z)-ethyl 2-((Z)-5-((dimethylamino) methylene)-4-oxo-3-phenylthiazolidin-2-ylidene)acetate (**6**) (Scheme 1). Spectral data (IR, NMR, and X-ray were in a complete agreement with the proposed structure.

**Scheme 2:**
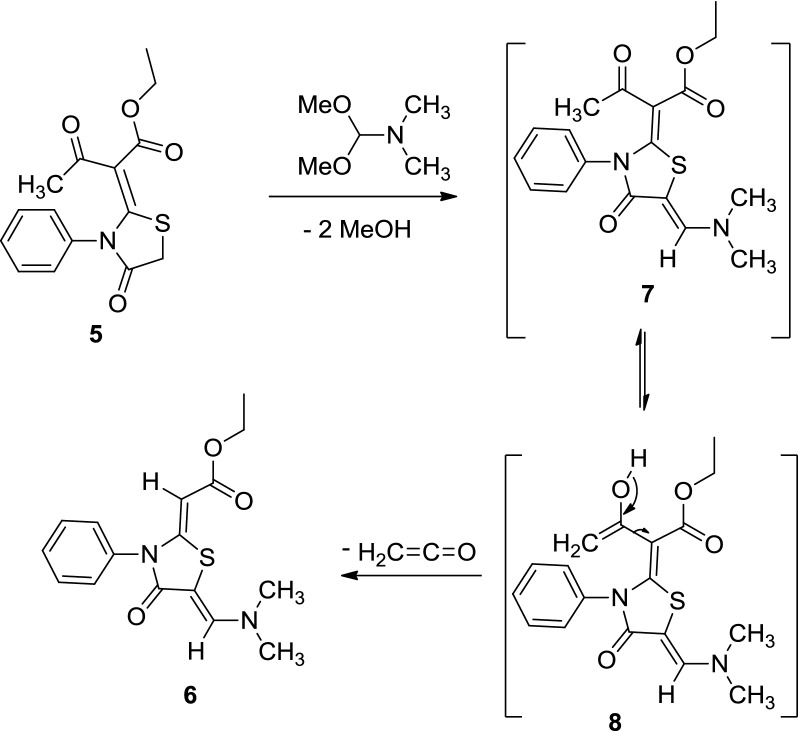
The suggested mechanism for the synthesis of thiazolidinone derivative **6**

**Fig. 2:**
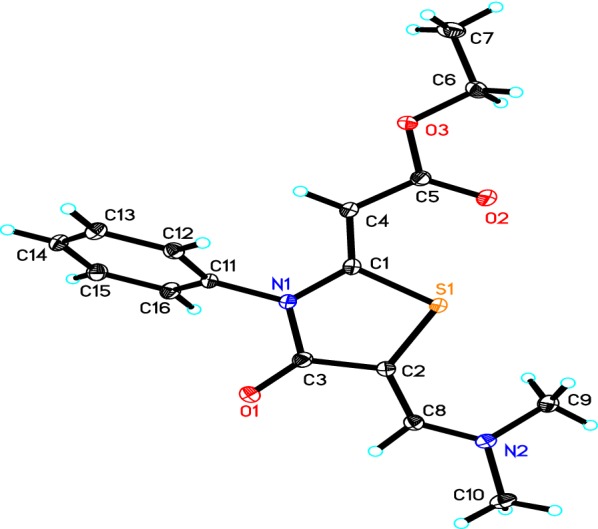
The ORTEP diagram of the final X-ray model of thiazolidinone **6** with displacement ellipsoids drawn at 50% probability level. H-atoms were placed and not included in refinement

The following reaction mechanism was suggested for the formation of thiazolidinone derivative **6** (Scheme 2). We assumed that the reaction was started between thiazolidinone **5** and dimethylformamide-dimethylacetal to produce an intermediate **7**, which underwent enolization of the carbonyl of the acetyl group, followed by elimination of ketene to give the thiazolidinone derivative **6** (Scheme 2). The configuration of thiazolidinone **6** was confirmed using X-ray analysis (Fig. 2) [16].

### Crystal structure determination

**Fig. 3:**
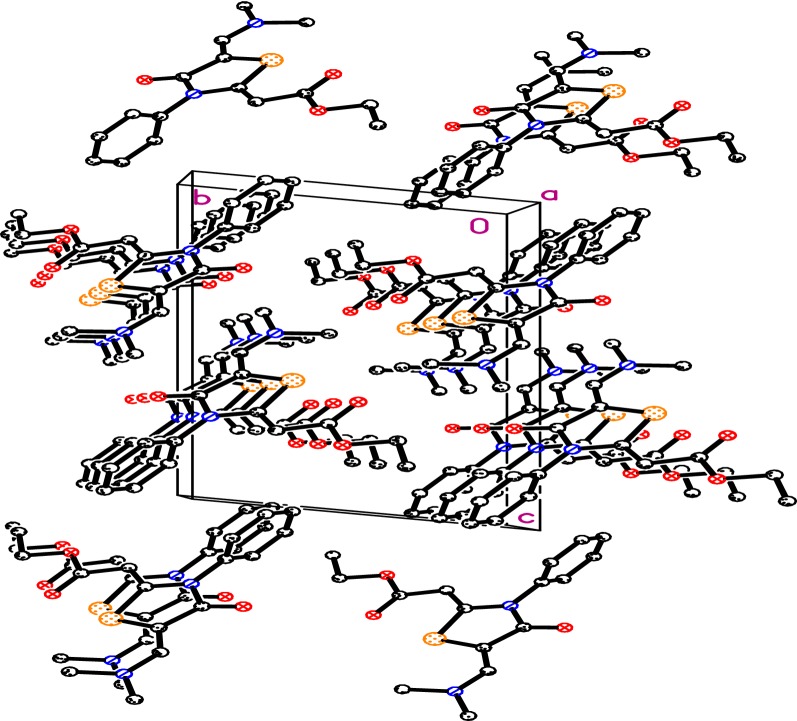
Molecular packing of the thiazolidinone **6**

A crystal of dimensions 0.47 × 0.26 × 0.14 mm was selected for X-ray diffraction analysis. Data were collected on a Bruker APEX-II diffractometer equipped with CCD detector and graphite monochromatic Mo Kα radiation (λ = 71,073 Å) at 100 °K. SHELXS-97 [17, 18] was used to solve structure (Table 1). Cell refinement and data reduction were carried out by Bruker SAINT [19]. The final refinement was carried out by full-matrix least-squares techniques with anisotropic thermal data for nonhydrogen atoms on F2. All the hydrogen atoms were placed in calculated positions. The crystal of thiazolidinone **6** (Fig. 2) was finally refined with R factor of 4.46% for 4622 unique reflections. Molecules were found to be packed in crystal lattice through intermolecular hydrogen bonding (Fig. 3). Table 2 summarized some selected geometric parameters for thiazolidinone 6.

**Table 1:** The crystal and experimental data of thiazolidinone **6**

Parameters	
Empirical formula	C_16_H_18_N_2_O_3_S
Formula weight	318.38
Temperature	100 °K
Wave length	0.71073 Å
Crystal system	Triclinic,
space group	P-1
Unit cell dimensions	a = 5.6502 (6) Å
	b = 9.1968 (9) Å
	c = 14.9469 (16) Å
	α = 98.992 (5)°
	β = 91.848 (7)°
	γ = 96.184 (5)°
Volume	761.73 (14) Å^3^
Z	2
Calculated density	1.388 Mg m^−3^
Absorption coefficient	0.23 mm^−1^
F(000)	336
Crystal size	0.47 × 0.26 × 0.14 mm
Theta range for data collection	1.4° to 30.7°
Limiting indices	− 8 ≤h ≤ 8, − 11 ≤k ≤ 13, − 21 ≤l ≤ 21
Reflections collected/unique	16,661/4622 [R(int) = 0.045]
Completeness to theta	30.7°–98.1%
Absorption correction	Semi-empirical from equivalents
Max. and min. transmission	0.7318 and 0.7106
Refinement method	Full-matrix least-squares on F-2
Data/restraints/parameters	4622/0/203
Goodness-of-fit on F-2	1.04
Final R indices [I > 2sigma(I)]	R1 = 0.0344, wR2 = 0.088
R indices (all data)	R1 = 0.0446, wR2 = 0.092
Largest diff. peak and hole	0.45 and − 0.32 e.Å^−3^

**Table 2:** Selected geometric parameters (Å, °) for thiazolidinone **6**

S1—C1	1.7461 (11)	N1—C1	1.3854 (14)
S1—C2	1.7634 (11)	N1—C3	1.4115 (14)
O1—C3	1.2261 (13)	N1—C11	1.4364 (13)
O2—C5	1.2219 (13)	N2—C8	1.3300 (14)
O3—C5	1.3560 (13)	N2—C9	1.4543 (15)
O3—C6	1.4535 (14)	N2—C10	1.4539 (15)
C1—S1—C2	91.59 (5)	S1—C2—C8	129.00 (8)
C5—O3—C6	114.88 (8)	O1—C3—N1	122.06 (9)
C1—N1—C3	116.76 (9)	O1—C3—C2	128.67 (10)
C1—N1—C11	122.46 (9)	N1—C3—C2	109.27 (9)
C3—N1—C11	120.76 (8)	O2—C5—O3	122.19 (10)
C8—N2—C9	122.19 (9)	O2—C5—C4	125.53 (10)
C8—N2—C10	121.08 (9)	O3—C5—C4	112.28 (9)
C9—N2—C10	116.31 (9)	O3—C6—C7	107.39 (9)
S1—C1—N1	110.58 (8)	N2—C8—C2	130.35 (10)
S1—C1—C4	124.14 (8)	N1—C11—C12	120.10 (9)
N1—C1—C4	125.27 (10)	N1—C11—C16	118.75 (9)
S1—C2—C3	111.73 (8)		

### Geometry optimization

**Fig. 4:**
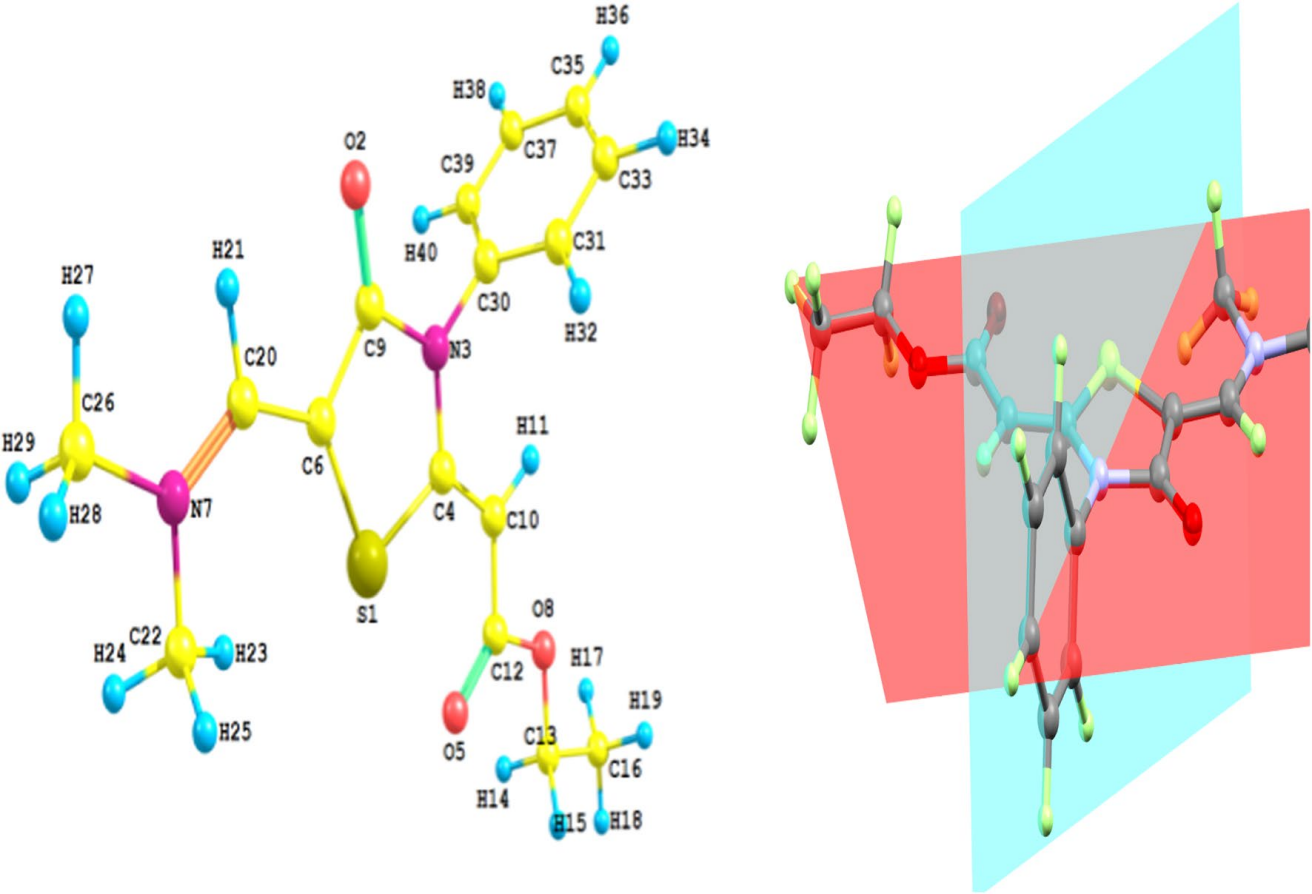
The optimized structure of thiazolidinone **6**

The optimized molecular geometry of thiazolidinone **6** is shown in Fig. 4. Table 3 listed the hydrogen bonding data for thiazolidinone **6**, and the results of the calculated bond distances and angles are given in Table 4. It is clear that the calculated bond distances and angles agree very well with the experimental results. The bond distances and angles deviated only by 0.027 Å (C20–N7) and 1.6° (C20–C6–S1). The correlation coefficients given in the same table indicated the good agreement between the calculated structure and the X-ray results. Interestingly, the thiazole and phenyl ring planes are not coplanar and are twisted from one another by an angle of 77.1° (exp. 66.5°).

**Table 3:** Hydrogen bonding data for thiazolidinone **6**

D—H···A	D—H	H···A	D···A	D—H···A
C8—H8···O1	0.95	2.53	2.8888 (13)	102
C9—H9A···S1	0.98	2.77	3.1190 (12)	102
C9—H9B···O2i	0.98	2.33	3.3018 (15)	171
C12—H12···O1ii	0.95	2.37	3.3042 (14)	168

Symmetry codes: (i) − x + 1, − y + 1, − z + 1; (ii) x − 1, y, z

**Table 4:** The experimental and calculated geometric parameters of thiazolidinone **6**

Bond distances	Calc.	X-ray	Bond angles	Calc.	X-ray
R(1–4)	1.773	1.746	A(4-1-6)	91.0	91.6
R(1–6)	1.786	1.763	A(1-4-3)	111.0	110.6
R(2–9)	1.223	1.226	A(1-4-10)	123.9	124.1
R(3–4)	1.387	1.385	A(1-6-9)	111.6	111.7
R(3–9)	1.417	1.412	A(1-6-20)	131.0	129.0
R(3–30)	1.435	1.436	A(2-9-3)	122.8	122.1
R(4–10)	1.362	1.361	A(2-9-6)	127.8	128.7
R(5–12)	1.227	1.222	A(4-3-9)	116.9	116.8
R(6–9)	1.463	1.444	A(4-3-30)	122.8	122.5
R(6–20)	1.363	1.369	A(3-4-10)	125.2	125.3
R(7–20)	1.357	1.330	A(9-3-30)	120.2	120.8
R(7–22)	1.456	1.454	A(3-9-6)	109.5	109.3
R(7–26)	1.456	1.454	A(3-30-31)	119.9	120.1
R(8–12)	1.360	1.356	A(3-30-39)	119.6	118.7
R(8–13)	1.441	1.453	A(4-10-12)	121.0	119.8
R(10–12)	1.448	1.444	A(5-12-8)	122.6	122.2
R(13–16)	1.517	1.506	A(5-12-10)	125.7	125.5
R(30–31)	1.397	1.391	A(9-6-20)	117.4	119.2
R(30–39)	1.396	1.388	A(6-20-7)	132.2	130.3
R(31–33)	1.394	1.391	A(20-7-22)	123.4	122.2
R(33–35)	1.396	1.389	A(20-7-26)	119.3	121.1
R(35–37)	1.396	1.390	A(22-7-26)	115.6	116.3
R(37–39)	1.394	1.390	A(7-22-23)	110.6	109.5
			A(7-22-24)	108.9	109.5
			A(12-8-13)	115.6	114.9
			A(8-12-10)	111.8	112.3
			A(8-13-16)	107.4	107.4
			A(11-10-12)	118.8	120.1
			A(31-30-39)	120.5	121.2
			A(30-31-33)	119.6	118.9
			A(30-39-37)	119.6	119.4
			A(32-31-33)	120.8	120.6
			A(31-33-35)	120.1	120.5
			A(33-35-37)	119.9	120.0
			A(35-37-39)	120.2	120.1
R2	0.9979			0.9936	

R2: correlation coefficient

### Charge population analysis

**Fig. 5:**
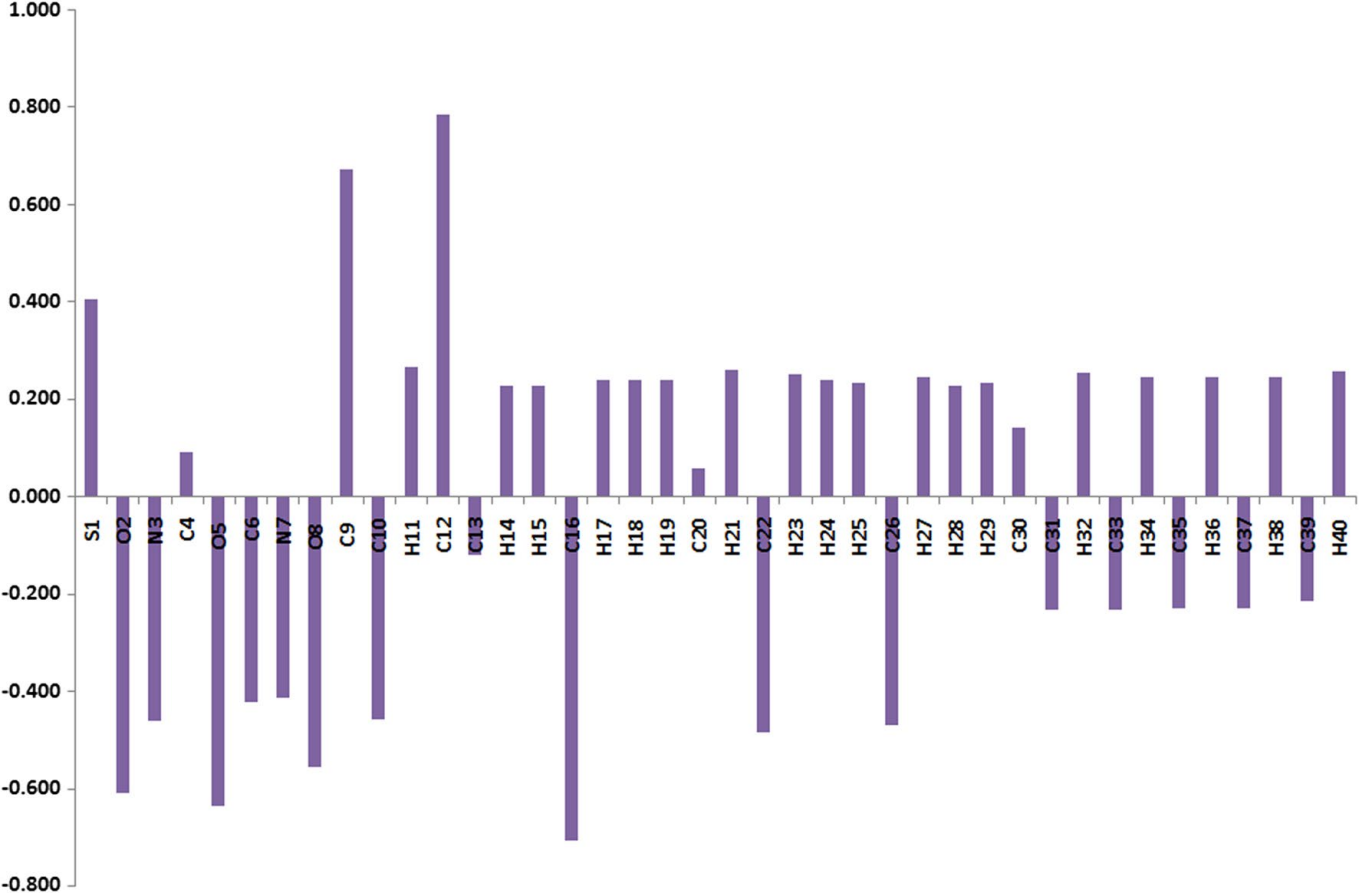
The natural charges at the different atomic sites of thiazolidinone **6**

**Fig. 6:**
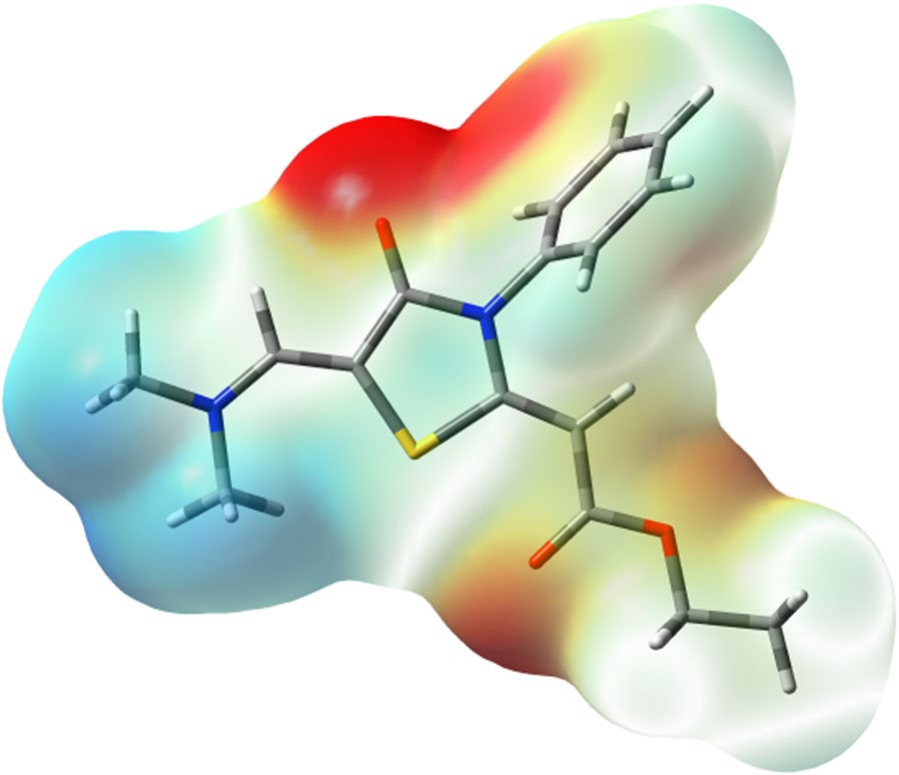
The MEP figure of thiazolidinone **6**

The natural population analysis is performed to predict the natural charges (NC) at the different atomic sites (Fig. 5). It is clear that the sulfur atom is electropositive. The C9 and C12 are the most electropositive C-atoms as these atomic sites are bonded to two strong electronegative atoms. In contrast, the O and N-atoms have electronegative nature where the ring N-atom (N3) is more electronegative than the imine one (N7). Among the O-atoms in the studied molecule, the carbonyl O-atoms are the most electronegative where the O-atom (O5) from the carbonyl group in the ester moiety is more negative than that of the cyclic ketone (O2). As revealed from the molecular electrostatic potential (MEP) map shown in Fig. 6, the most negative regions are located over the O-atoms while the positive region is mainly located over the protons of the two methyl groups.

### Frontier molecular orbitals

**Fig. 7:**
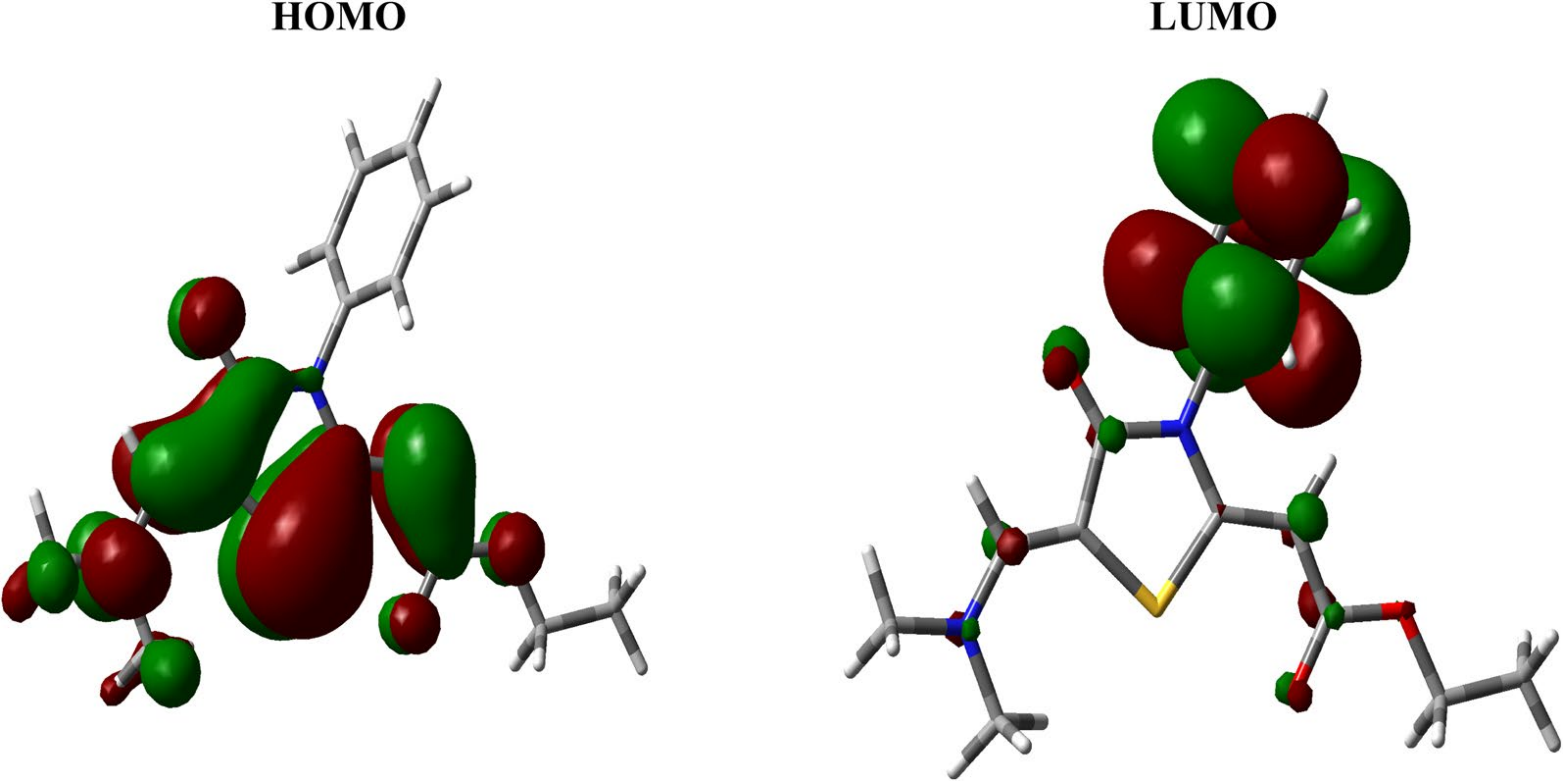
The frontier molecular orbitals calculated at the B3LYP/6-311G(d,p) level

The HOMO and LUMO levels of the thiazolidinone **6** are shown in Fig. 7. The HOMO and LUMO energies are − 5.0584 and − 0.9870 eV, respectively. As a result, the HOMO–LUMO energy gap is calculated to be 4.0714 eV. The HOMO and LUMO are mainly localized over the thiazole and phenyl rings, respectively. Since the HOMO and LUMO levels are mainly located over the π-system of the studied compound **6** so the HOMO–LUMO intramolecular charge transfer is mainly a π–π* transition.

### NMR spectra

**Fig. 8:**
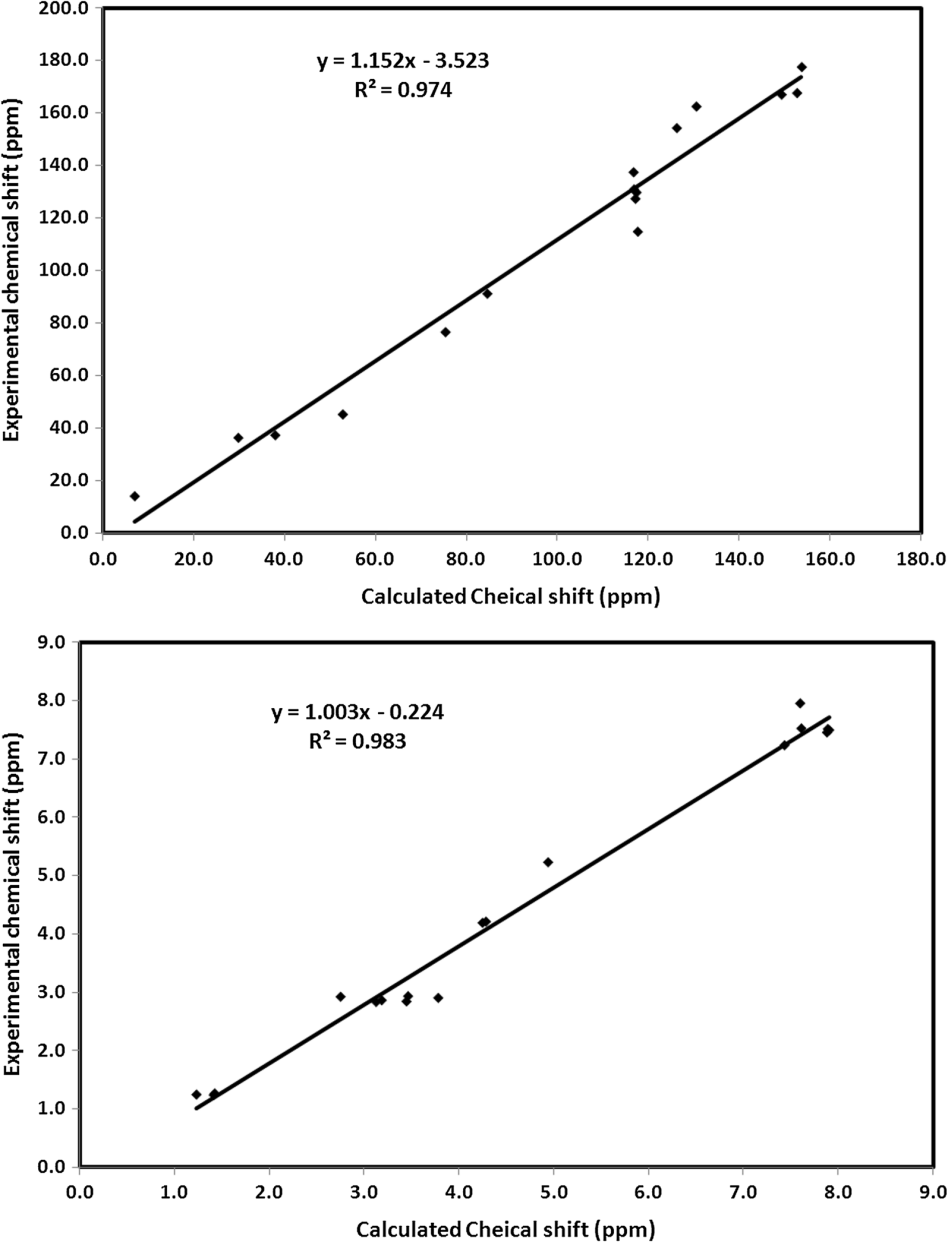
Correlation graphs between the calculated and experimental ^1^H and ^13^C NMR chemical shifts

The Gauge-independent atomic orbital (GIAO) calculations were used for accurate prediction of the ^1^H and ^13^C isotropic chemical shifts (C.S). The isotropic chemical shifts were used for the identification of organic compounds. The theoretical and experimental chemical shifts are presented in Table 5. Correlation graphs between the experimental and theoretical NMR chemical shifts are shown in Fig. 8. The correlations equations shown in this figure have high R2 values (0.974–0.983) indicating the good agreement between the theoretical and experimental data. The ^1^H-chemical shifts of the aromatic ring usually appear in the region of 7–8 ppm. In the present case, the aromatic protons were detected at 7.46–7.96 ppm which is in good agreement with B3LYP theoretical values (7.60–7.89 ppm). The aliphatic protons have lower chemical shifts than the aromatic ones (Table 5).

**Table 5:** The calculated and experimental ^1^H and ^13^C NMR chemical shifts of thiazolidinone **6**

Atom	C. Scalc.	C. Sexp.	Atom	C. Scalc.	C. Sexp.
C4	149.2	166.9	H11	4.94	5.23
C6	84.5	91.2	H14	4.25	4.19
C9	152.6	167.5	H15	4.29	4.21
C10	75.3	67.5	H17	1.41	1.23
C12	153.7	177.4	H18	1.23	1.24
C13	52.7	60.9	H19	1.42	1.26
C16	6.9	14.3	H21	7.44	7.24
C20	130.5	162.4	H23	3.78	2.90
C22	29.7	36.4	H24	2.75	2.92
C26	37.8	37.4	H25	3.46	2.93
C30	126.2	154.2	H27	3.13	2.83
C31	116.8	130.9	H28	3.45	2.84
C33	117.1	127.3	H29	3.19	2.86
C35	116.7	137.5	H32	7.61	7.53
C37	117.3	129.7	H34	7.89	7.52
C39	117.6	114.8	H36	7.88	7.46
			H38	7.91	7.50
			H40	7.60	7.96

The chemical shifts of the aromatic carbons usually appear in the overlapped region of the spectrum between 100 and 200 ppm [20]. The atom C30 has higher chemical shift than the other aromatic carbons (Table 5). The high chemical shift of C30 is due to the deshielding effect of the electronegative N-atom. Since the oxygen and nitrogen atoms are more electronegative than carbons so the C-atoms (C4, C9, C12, and C20) attached to these electronegative sites were detected at high chemical shifts (162.4–177.4 ppm) compared to the rest of C-atoms.

### Cytotoxic activity

The anti-cancer activity of the thiazolidine derivatives **5** and **6** was determined against the colon carcinoma (HCT-116) cell line in comparison with the anticancer drug vinblastine, using MTT assay [21]. The results of the cytotoxic activity were expressed as the mean IC_50_ of three independent experiments (Table 6). The results revealed that thiazolidinone derivatives **5** and 6 had moderate anticancer activity against colon carcinoma (HCT-116).

**Table 6:** Viability values and IC_50_ of thiazolidinone derivatives **5** and **6** against HCT-116 Cell Line

S. No	Sample concentration (μg/mL) viability %							
	50	25	12.5	6.25	3.125	1.56	0	IC_50_ (μg)
Vinblastine	23.08	27.35	43.59	53.85	69.23	82.54	100	9.8
**5**	42.51	76.82	84.19	93.72	98.56	100	100	44.5
**6**	39.43	58.15	79.51	86.42	92.63	96.47	100	35.9

## Experimental section

### Chemistry

#### General

All the melting points were measured on a Gallenkamp apparatus in open glass capillaries and are uncorrected. The IR Spectra were recorded using Nicolet 6700 FT-IR spectrophotometer. ^1^H- and ^13^C-NMR spectra were recorded on a JEOL ECP 500 NMR spectrometer operating at 500 MHz. ^1^H spectra were run at 500 MHz and ^13^C spectra were run at 125 MHz in deuterated chloroform (CDCl_3_). Chemical shifts were related to that of the solvent. Chemical shifts δ are expressed in ppm units. Elemental analysis were carried out on a 2400 CHN Elemental Analyzer. The single-crystal X-ray diffraction measurements were accomplished on a Bruker SMART APEX II CCD diffractometer. The biological evaluations of the products were carried out in the Medical Mycology Laboratory of the Regional Center for Mycology and Biotechnology of Al-Azhar University, Cairo, Egypt. The thiazolidinone derivative **5** was prepared as described in the literature [15].

##### Synthesis of (Z)-ethyl 2-((Z)-5-((dimethylamino)methylene)- 4-oxo-3-phenylthiazolidin- 2-ylidene)acetate (6)

A mixture of thiazolidinone **5** (3.05 g, 10 mmol) and DMF-DMA (1.19 g, 1.33 mL, 10 mmol) in DMF (3 mL) was heated under reflux for 3 h, then left to cool to room temperature. The precipitated solid was filtered off, washed with EtOH and recrystallized from DMF to afford the thiazolidinone derivative **6** in 20% yield, m.p. 227 °C; IR (KBr) v max 1715 (C=O), 1639 (C=O) cm^−1^; ^1^H NMR (500 MHz, CDCl_3_): δ 1.24 (t, 3H. CH_3_, *J *= 7.5 Hz), 2.83 (s, 3H, CH_3_), 2.91 (s, 3H, CH_3_), 4.20 (q, 2H, CH_2_, *J *= 7.5 Hz), 5.23 (s, 1H, CH), 7.24 (s, 1H, CH), 7.38–7.96 (m, 5H, Ar–H); ^13^C NMR (125 MHz, CDCl_3_): δ 14.3 (CH_3_), 36.4, 37.4 (2CH_3_), 60.9 (CH_2_), 67.5, 91.2, 114.8, 127.3, 129.7, 130.9, 137.5, 154.2, 162.4, 166.9, 167.5 (C=O), 177.4 (C=O); MS, *m*/*z* (%) 304 (95), 258 (55), 243 (100), 215 (25), 77 (Ph, 18). calcd. for C_16_H_18_N_2_O_3_S: C, 60.36; H, 5.70; N, 8.80. Found: C, 60.28; H, 5.66; N, 8.85.

## X-ray analysis

The thiazolidinone **6** was obtained as single crystals by slow evaporation from DMF solution of the pure compound at room temperature. The crystallographic data for thiazolidinone **6** (CCDC 1551169) can be obtained on request from the director, Cambridge Crystallographic Data Center, 12 Union Road, Cambridge CB2 1EW, UK http://www.ccdc.cam.ac.uk/data_request/cif.

### Computational details

The X-ray structure coordinates of thiazolidinone **6** were used for geometry optimization followed by frequency calculations. For this task, we used Gaussian 03 software [22] and B3LYP/6‒31G(d,p) method. All the obtained frequencies are positive and no imaginary modes were detected. GaussView4.1 [23] and Chemcraft [24] programs have been used to extract the calculation results and to visualize the optimized structures.

### Cytotoxic activity

The cytotoxic activity of the synthesized compounds was carried out at the Regional Center for Mycology and Biotechnology at Al-Azhar University, Cairo, Egypt according to the reported method [21].

## Conclusions

A novel synthesis and DFT studies of (Z)-ethyl 2-((Z)-5-((dimethylamino) methylene)-4-oxo-3-phenylthiazolidin-2-ylidene)acetate are presented. The NMR chemical shifts were described based on the GIAO calculations. The calculated results showed good correlations with the experimental data. The anticancer activity of the synthesized compounds against the colon carcinoma (HCT-116) cell line was tested and showed moderate activity.
